# Factors Contributing to the Development of Neuropsychiatric Manifestations in Persons With Multiple Sclerosis: A Systematic Review

**DOI:** 10.7759/cureus.66432

**Published:** 2024-08-08

**Authors:** Elizabeth Sangster, Nidhi Lanka, Prakash Acharya, Shikha Virani, Sumayya Afreen, Arvin Perthiani, Sondos T Nassar

**Affiliations:** 1 Psychiatry and Behavioral Sciences, California Institute of Behavioral Neurosciences & Psychology, Fairfield, USA; 2 School of Medicine, St. George's University, St. George's, GRD; 3 General Medicine, California Institute of Behavioral Neurosciences & Psychology, Fairfield, USA; 4 General Medicine, Patan Academy of Health Sciences, Lalitpur, NPL; 5 Medicine, Surat Municipal Institute of Medical Education and Research, Surat, IND; 6 General Practice, Deccan College of Medical Sciences, Hyderabad, IND; 7 Obstetrics and Gynecology, California Institute of Behavioral Neurosciences & Psychology, Fairfield, USA; 8 General Surgery, Our Lady of Lourdes Hospital, Drogheda, Drogheda, IRL; 9 General Surgery, California Institute of Behavioral Neurosciences & Psychology, Fairfield, USA; 10 Medicine and Surgery, Jordan University of Science and Technology, Amman, JOR

**Keywords:** mental health, mood disorder, neurodegeneration, cognitive impairment, risk factors, quality of life, anxiety, depression, neuropsychiatric symptoms, multiple sclerosis

## Abstract

Multiple sclerosis (MS) is the most common chronic demyelinating disease affecting the central nervous system (CNS) and is distinguished by neuroinflammation and neurodegeneration. It has four categories based on clinical course, with relapsing-remitting being the most common type. MS predominantly manifests with motor and sensory dysfunctions. However, neuropsychiatric manifestations such as depression, anxiety, schizophrenia, and bipolar disorder are not uncommon. Various factors may contribute to the development of these manifestations; therefore, this study aimed to unravel them.

This systematic review implemented the Preferred Reporting Items for Systematic Reviews and Meta-Analyses (PRISMA) 2020 guidelines. Five databases (PubMed, PubMed Central (PMC), ScienceDirect, Cochrane Library, and Google Scholar) were used to acquire articles published in the past five years. After screening and quality appraisal were completed, eight articles were deemed eligible for inclusion in this study. The study designs included cohort, cross-sectional, randomized-controlled trial (RCT), case report, case-control, and narrative review. The development of neuropsychiatric manifestations in persons with MS is influenced by various factors. These were categorized into morphological changes of the brain, immunological mechanisms, socioeconomic factors, and individual factors for discussion. Each factor was found to intermingle with the others, requiring further research to understand the features that each factor contributes. This is crucial for improving the quality of life (QOL) and prognosis for persons living with MS.

## Introduction and background

Being the most prevalent demyelinating disease of the central nervous system (CNS) [1], multiple sclerosis (MS) is characterized by immune dysregulation that causes myelin loss and axonal degeneration [2]. There are four categories of MS based on the clinical course of the illness: relapsing-remitting, secondary progressive, primary progressive, and progressive-relapsing, with relapsing-remitting being the predominant type [3]. Damage can occur anywhere in the CNS, but the white matter tracts in the optic nerves, cerebral hemispheres, brain stem, cerebellum, and spinal cord are primarily affected [3]. Common clinical manifestations include impaired vision, bowel/ bladder dysfunction, numbness, cognitive impairment, and psychiatric symptoms [4].

MS is also the most common neuroinflammatory disease that causes neurological non-traumatic disability among young adults, affecting women aged 20 to 40 years the most [4]. Moreover, because of its devastatingly chronic and progressive nature, MS is associated with high physical, emotional, and cognitive morbidity, which can impact a person’s overall health and well-being [2,4]. This makes the manifestation of neuropsychiatric symptoms common and may be categorized into disorders of mood, affect, and behavior, and abnormalities of cognition [5]. Research over the years has indicated that some of these symptoms may have existed in patients before the diagnosis of MS, but the majority of cases have indicated that they were developed along the course of the disease progression. Depression is highlighted as the most common neuropsychiatric manifestation, followed by anxiety disorders [4]. It is, however, not uncommon for persons with MS to also have schizophrenia and/or bipolar disorder [2].

Several studies tried to establish a relationship between neuropsychiatric symptoms and MS based on risk factors, severity of the disease course, and impact on quality of life (QOL) [6]; however, no definitive consensus was made [1]. Furthermore, these neuropsychiatric conditions may influence how persons with MS cope with stressors, seek medical support, carry out daily activities of living, and prognosis [7]. Hence, the goal of this study was to determine which factors contribute to the development of neuropsychiatric symptoms in persons with any form of MS to better understand and improve management for persons living with MS.

## Review

Methods

The Preferred Reporting Items for Systematic Reviews and Meta-Analyses (PRISMA) 2020 [8] guidelines were followed to complete this systematic review.

Search Strategy

The systematic search focused on articles published between January 1, 2020, to May 12, 2024. Five databases were used in the search: PubMed, PubMed Central (PMC), Science Direct, Cochrane Library, and Google Scholar. The keywords utilized during the search were multiple sclerosis and neuropsychiatric symptoms or psychiatric conditions or psychiatric symptoms. Based on the database, the field search encompassed either keyword boolean or medical subject headings (MeSH). Table 1 provides the search strategy implemented. 

**Table 1:** Search strategy and results for the five databases PMC: PubMed Central

Database	Keywords	Search strategy	Number of articles before filters	Filters	Search result
PubMed	Multiple sclerosis, Neuropsychiatric symptoms or psychiatric conditions or psychiatric symptoms	("multiple sclerosis"[MeSH Terms] OR ("multiple"[All Fields] AND "sclerosis"[All Fields]) OR "multiple sclerosis"[All Fields]) AND ((y_5[Filter]) AND (ffrft[Filter])) ((("neuropsychiatric"[All Fields] OR "neuropsychiatrically"[All Fields] OR "neuropsychiatrics"[All Fields]) AND ("diagnosis"[MeSH Subheading] OR "diagnosis"[All Fields] OR "symptoms"[All Fields] OR "diagnosis"[MeSH Terms] OR "symptom"[All Fields] OR "symptom s"[All Fields] OR "symptomes"[All Fields])) OR (("psychiatrical"[All Fields] OR "psychiatrically"[All Fields] OR "psychiatrics"[All Fields] OR "psychiatry"[MeSH Terms] OR "psychiatry"[All Fields] OR "psychiatric"[All Fields]) AND ("diagnosis"[MeSH Subheading] OR "diagnosis"[All Fields] OR "symptoms"[All Fields] OR "diagnosis"[MeSH Terms] OR "symptom"[All Fields] OR "symptom s"[All Fields] OR "symptomes"[All Fields])) OR ("mental disorders"[MeSH Terms] OR ("mental"[All Fields] AND "disorders"[All Fields]) OR "mental disorders"[All Fields])) AND ((y_5[Filter]) AND (ffrft[Filter])) (("multiple sclerosis"[MeSH Terms] OR ("multiple"[All Fields] AND "sclerosis"[All Fields]) OR "multiple sclerosis"[All Fields]) AND ("2020/04/29 00:00":"3000/01/01 05:00"[Date - Publication] AND "loattrfree full text"[Filter]) AND ((("neuropsychiatric"[All Fields] OR "neuropsychiatrically"[All Fields] OR "neuropsychiatrics"[All Fields]) AND ("diagnosis"[MeSH Subheading] OR "diagnosis"[All Fields] OR "symptoms"[All Fields] OR "diagnosis"[MeSH Terms] OR "symptom"[All Fields] OR "symptom s"[All Fields] OR "symptomes"[All Fields])) OR (("psychiatrical"[All Fields] OR "psychiatrically"[All Fields] OR "psychiatrics"[All Fields] OR "psychiatry"[MeSH Terms] OR "psychiatry"[All Fields] OR "psychiatric"[All Fields]) AND ("diagnosis"[MeSH Subheading] OR "diagnosis"[All Fields] OR "symptoms"[All Fields] OR "diagnosis"[MeSH Terms] OR "symptom"[All Fields] OR "symptom s"[All Fields] OR "symptomes"[All Fields])) OR ("mental disorders"[MeSH Terms] OR ("mental"[All Fields] AND "disorders"[All Fields]) OR "mental disorders"[All Fields])) AND ("2020/04/29 00:00":"3000/01/01 05:00"[Date - Publication] AND "loattrfree full text"[Filter])) AND ((y_5[Filter]) AND (ffrft[Filter]))	116810	Articles from 2020 to 2024, free full texts, human studies, English language, all research types	1021 manually removed-
Google Scholar	Multiple sclerosis, psychiatric conditions	Keyword Boolean: "Multiple Sclerosis" AND “Neuropsychiatric	16900	Articles from 2020 to 2024, all research types	973
Science Direct	Multiple sclerosis, Neuropsychiatric symptoms	Keyword Boolean: "Multiple Sclerosis" AND “psychiatric symptoms”	2639	Articles from 2020 to 2024, free access, all research types	184
PMC	Multiple sclerosis, Neuropsychiatric symptoms	Keyword Boolean: "Multiple Sclerosis" AND “Neuropsychiatric symptoms”	3033	Articles from 2020 to 2024, free access, all research types	1760
Cochrane Library	Multiple sclerosis, Neuropsychiatric symptoms	Keyword Boolean: "Multiple Sclerosis" AND “Neuropsychiatric symptoms” OR “psychiatric symptoms” OR “Mental disorders”	15379	Articles from 2020 to 2024, full-texts only, research types: trials and reviews	54
Total					3 992

Study Selection

After articles were gathered and imported into the EndNote 21 application (Clarivate PLC; Philadelphia, United States; London, United Kingdom), the authors screened the titles and abstracts to identify suitable articles for full-text review. The selection process utilized pre-defined inclusion and exclusion criteria. Table 2 highlights the inclusion and exclusion criteria.

**Table 2:** Inclusion and exclusion criteria utilized in the study selection QOL: quality of life; MS: multiple sclerosis

Inclusion criteria	Exclusion criteria
Study period: five years (January 1, 2020, to May 12, 2024)	Studies outside of the study period
Human studies	Animal studies
English original or translated articles	Languages other than English
Free full texts	Patients with other neurogenerative and demyelinating diseases
Patients with MS and neuropsychiatric symptoms	Patients without MS and neuropsychiatric symptoms
Any patient population or age range with MS and neuropsychiatric symptoms	Studies about treatment protocols/ regimens for other non-neuropsychiatric symptoms of MS.
Studies about cognition performance in persons with MS	
Studies pertaining to QOL in persons with MS	

Data Collection and Analysis

Articles were gathered from the five stated databases. Study selection criteria were applied, and full articles were retrieved for further review. The articles were then categorized by study design and primarily included studies of randomized controlled trials (RCTs), systematic reviews, narrative reviews, case-control studies, cohort studies, cross-sectional studies, and case reports. Further, details retrieved from articles included title, author name, publication date, and information relevant to the research aim. Respective quality assessment tools were also utilized for the different study design categories to screen for bias.

After assessing the risk of bias, the accepted articles were categorized into discussion topics that reflected the factors contributing to the development of neuropsychiatric manifestations in persons with MS. These are summarized in the results section.

Results

**Figure 1:**
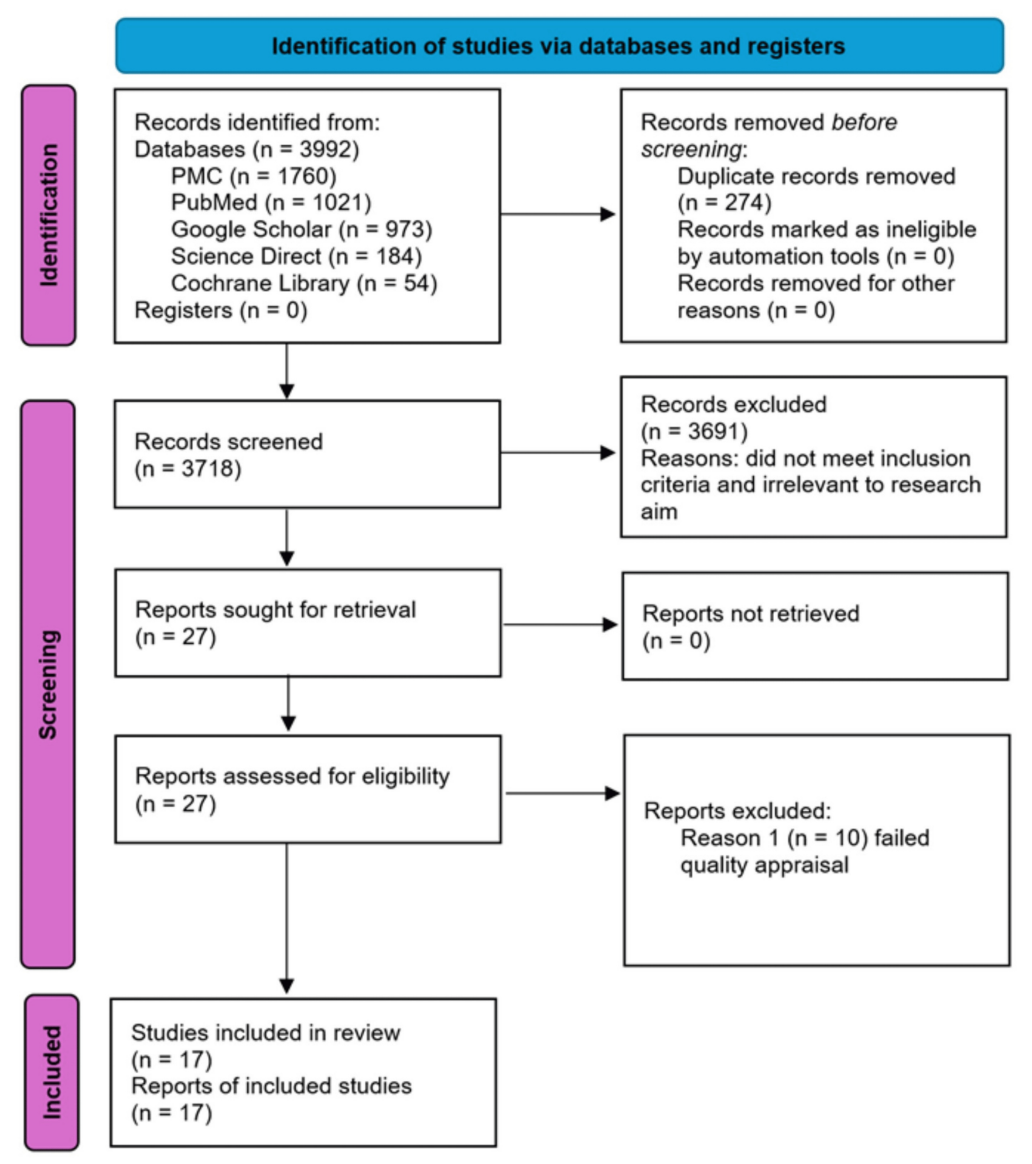
PRISMA 2020 flow diagram illustrating the screening process for systematic review PMC: PubMed Central; PRISMA: Preferred Reporting Items for Systematic Reviews and Meta-Analyses

A total of 3992 articles were gathered from the five main databases. An initial screening for duplicates was done, and 274 duplicate articles were removed. The remaining 3718 articles were further reviewed for inclusion or exclusion to the study. This was based on inclusion/exclusion criteria and relevance to the research aim by reviewing the abstract, method, result, and discussion. A total of 3691 articles were subsequently removed due to not fulfilling the necessary requirements of the research aim or inclusion criteria. The remaining 27 articles were assessed for quality appraisal; 10 failed and were excluded from the study. Figure 1 summarizes these details using the PRISMA flow diagram [8].

Risk of Bias Assessment

After careful screening, retrieval of articles, and categorization of study designs, the remaining articles were processed through their respective quality appraisal tools. Those deemed acceptable were used in this systematic review. Each quality assessment tool consisted of specific signaling questions/criteria and accompanying response options that were scored. For articles to be acceptable for use in this study, a score of ≥70% had to be made on their respective quality assessment tool, and they were marked as “pass.” The studies that did not make the passing score were marked as “fail” and rejected for use. The tables below summarize the articles assessed, the respective quality appraisal tools implemented, signaling questions/criteria used, and their outcomes.

Table 3 summarizes the appraisal tools, their respective criteria utilized for quality assessment, and the number of studies that passed or failed. 

**Table 3:** Summarizing the quality appraisal tool utilized for each study design and the number of studies assessed PICO: patient/population, intervention, comparison, and outcomes

Appraisal tool and study design	Signaling question/criteria utilized for appraisal	Number of studies assessed	Number passed	Number failed
The New Castle-Ottawa (NOS) quality assessment scale; cohort studies	The following criteria were used to assess the articles: ensuring the representativeness of the exposed cohort in the community - this includes assessing if the cohort is truly representative or somewhat representative of the average individual in the community; selecting the non-exposed cohort from the same community as the exposed cohort; ascertaining exposure through secure records or structured interviews; demonstrating that the outcome of interest was not present at the beginning of the study; comparing the cohorts based on the study design or analysis and controlling for the most important factors, as well as any additional factors; assessing the outcome and ensuring that the follow-up period is long enough for outcomes to occur; and adequately following up with cohorts, which involves accounting for all subjects and addressing any potential bias introduced by subjects lost to follow-up.	4	3	1
The NOS quality assessment scale; case-control studies	The following criteria were used to assess the articles: case definition adequacy; case representativeness; control definition and selection; comparability of cases and controls based on the design or analysis; methods of ascertainment of exposure for cases and controls; and rate of non-response in the study.	5	3	2
The Joanna Briggs Institute (JBI) critical appraisal checklist; case reports	The following criteria were used to assess the articles: patient demographic characteristics; patient history as a timeline; clinical condition upon presentation; description of diagnostic tests or assessment methods and results; intervention or treatment details; post-intervention clinical condition; identification and description of adverse events or unanticipated event; and takeaway lessons from the case report.	2	1	1
The Revised Tool to assess Risk of Bias in randomized trials (RoB2); randomized-controlled trials	The following criteria were used to assess the articles: criteria 1 focuses on the random allocation sequence and baseline differences between groups; criteria 2 involves participant and provider awareness of the intervention, deviations from the intended intervention, and the impact of these deviations on the outcome; criteria 3 emphasizes the availability of outcome data for all participants and potential biases resulting from missing data; criteria 4 pertains to the measurement and assessment of outcomes, including the assessor's awareness of the intervention's impact; and criteria 5 stresses the importance of following a pre-specified analysis plan and avoiding result selection bias.	2	1	1
The Appraisal Tool for Cross-Sectional Studies (AXIS); cross-sectional studies	The following criteria were used to assess the articles: clear articulation of study aims and objectives; appropriateness of the study design in relation to the stated aims; justification of the sample size; clearly defined target/reference population; appropriateness of the sample frame in representing the target/reference population; selection process to ensure representativeness of subjects/participants; measures addressing and categorizing non-responders; appropriate measurement of risk factor and outcome variables; precision of statistical significance and estimates determination; sufficient description of methods for reproducibility; adequate description of basic data; consideration of non-response bias due to response rate; description of non-responders (if applicable); internal consistency of results; presentation of results for all described analyses; justification of author's discussions and conclusions by the results; discussion of study limitations; and disclosure of funding sources or conflicts of interest.	7	5	2
The Scale for the Assessment of Narrative Review Articles (SANRA); narrative reviews	The following criteria were utilized to assess the articles: rationale for the article's significance to the audience; clear aims and research questions; outline of literature search; inclusion of references; scientific reasoning and justification; and appropriateness of data presentation.	5	4	1
Assessing the Methodological Quality of Systematic Reviews (AMSTAR); systematic reviews	The following criteria were utilized to assess the articles: inclusion of PICO components in the research aims and inclusion criteria; explicit explanation of review methods and justification of any deviations from the protocol in the report; explanation of study selection; explanation of the use of search strategy and data extraction; performance of data extraction in duplicate by review authors; provision of a list of excluded studies and a detailed description of included studies; assessment of bias; reporting of funding sources; and addressing potential conflicts of interest by review authors.	2	0	2

Table 4 summarizes the articles that passed and were included in the discussion.

**Table 4:** Summary of passed and included articles along with respective quality assessment tools used NOS: New Castle-Ottawa; AXIS: Appraisal Tool for Cross-Sectional Studies; JBI: Joanna Briggs Institute; RoB2: Revised Tool to assess Risk of Bias in Randomized Trials; SANRA: Scale for the Assessment of Narrative Review Articles

Author, year	Study title	Study design	Quality appraisal tool utilized
Mustač et al., 2021 [1]	Anxiety and depression as comorbidities of multiple sclerosis	Narrative review	SANRA
Meier et al., 2020 [2]	Risk of schizophrenia and bipolar disorder in patients with multiple sclerosis: record-linkage studies	Cohort study	NOS for cohort studies
Alswat et al., 2023 [4]	The prevalence of major depression and generalized anxiety disorder in patients with multiple sclerosis in Saudi Arabia: a cross-sectional multicentered study	Cross-sectional study	AXIS critical appraisal of cross-sectional studies
Margoni et al., 2023 [5]	Depressive symptoms, anxiety and cognitive impairment: emerging evidence in multiple sclerosis	Narrative review	SANRA
Bruno et al., 2020 [9]	Inflammation-associated synaptic alterations as shared threads in depression and multiple sclerosis	Narrative review	SANRA
Alhussain et al., 2021 [10]	The relationship between vitamin D levels and cognitive impairment in patients with multiple sclerosis	Cross-sectional study	AXIS critical appraisal of cross-sectional studies
Ahmed et al., 2022 [11]	Cognitive impairment in paediatric onset multiple sclerosis and its relation to thalamic volume and cortical thickness of temporal lobe by magnetic resonance imaging	Case-control study	NOS for case-control studies
Oset et al., 2020 [12]	Cognitive dysfunction in the early stages of multiple sclerosis-how much and how important?	Narrative review	SANRA
Adamaszek et al., 2022 [13]	Clinical and neurophysiological patterns of impairments to emotion attention and empathy in multiple sclerosis	Case-control study	NOS for case-control studies
Almulla et al., 2023 [14]	Mood symptoms and chronic fatigue syndrome due to relapsing-remitting multiple sclerosis are associated with immune activation and aberrations in the erythron	Case-control study	NOS for case-control studies
Chan et al., 2021 [15]	Depression in multiple sclerosis across the adult lifespan	Cross-sectional study	AXIS critical appraisal of cross-sectional studies
Biasi et al., 2023 [16]	Impact of depression on the perception of fatigue and information processing speed in a cohort of multiple sclerosis patients	Cross-sectional study	AXIS critical appraisal of cross-sectional studies
Cuerda-Ballester et al., 2023 [17]	Relationship of motor impairment with cognitive and emotional alterations in patients with multiple sclerosis	Cross-sectional study	AXIS critical appraisal of cross-sectional studies
Marrie et al., 2021 [18]	Higher Framingham risk scores are associated with greater loss of brain volume over time in multiple sclerosis	Cohort study	NOS for cohort studies
Podda et al., 2020 [19]	Predictors of clinically significant anxiety in people with multiple sclerosis: a one-year follow-up study	Cohort study	NOS for cohort studies
Polick et al., 2021 [20]	The importance of assessing life stress exposure in multiple sclerosis: a case report	Case report	JBI for case reports
Nauta et al., 2023 [21]	Cognitive rehabilitation and mindfulness reduce cognitive complaints in multiple sclerosis (REMIND-MS): a randomized controlled trial	Randomized controlled trial	RoB2

Table 5 summarizes the articles that failed and were excluded from the discussion.

**Table 5:** Summary of failed and excluded articles along with respective quality assessment tools used NOS: New Castle-Ottawa; AXIS: Appraisal Tool for Cross-Sectional Studies; JBI: Joanna Briggs Institute; RoB2: Revised Tool to assess Risk of Bias in Randomized Trials; SANRA: Scale for the Assessment of Narrative Review Articles; AMSTAR: Assessing the Methodological Quality of Systematic Reviews

Author, Year	Study Title	Study Design	Quality Appraisal Tool Utilized
Alharbi et al., 2022 [6]	Prevalence of depression and anxiety among adult patients with multiple sclerosis at Riyadh City, Saudi Arabia	Cross-sectional study	AXIS Critical Appraisal of Cross-Sectional Studies
Barak-Corren et al., 2023 [22]	Improving risk prediction for target subpopulations: predicting suicidal behaviors among multiple sclerosis patients	Cohort study	NOS for cohort studies
Baller et al., 2023 [23]	Mapping the relationship of white matter lesions to depression in multiple sclerosis	Case-Control study	NOS for case-control studies
Cote et al., 2023 [24]	Caudate volume and symptoms of apathy in older adults with multiple sclerosis	Case-Control study	NOS for case-control studies
Reza et al., 2023 [25]	Neuropsychiatric manifestations of multiple sclerosis and the effects of modern disease-modifying therapies	Case report	JBI for case reports
Bijani et al., 2022 [26]	The effect of peer education based on Pender’s health promotion model on quality of life, stress management and self-efficacy of patients with multiple sclerosis: a randomized controlled clinical trial	Randomized controlled trial	RoB2
Meyer-Arndt et al., 2020 [27]	Neural processes of psychological stress and relaxation predict the future evolution of quality of life in multiple sclerosis	Cross-sectional study	AXIS Critical Appraisal of Cross-Sectional Studies
Cocchi et al., 2022 [28]	The inflammatory conspiracy in multiple sclerosis: a crossroads of clues and insights through mast cells, platelets, inflammation, gut microbiota, mood disorders and stem cells	Narrative review	SANRA
Concerto et al., 2021 [29]	Vitamin D and depressive symptoms in adults with multiple sclerosis: a scoping review	Systematic review	AMSTAR
Joseph et al., 2021 [30]	Prevalence of bipolar disorder in multiple sclerosis: a systematic review and meta-analysis	Systematic review	AMSTAR

Outcomes

Looking at the qualified articles for this systematic review, the outcomes and relevant information to address the factors contributing to neuropsychiatric symptoms' development in persons with MS can be categorized broadly into morphological, immunological, socioeconomic, and demographical factors. These components are explained in the discussion.

Discussion

Multiple sclerosis is known for being a debilitating disease of the central nervous system (CNS). Much research has been done to assess various factors that contribute to its development as well as the management of its course. The primary manifestations of MS are motor- and sensory-related; however, it is not uncommon for cognitive impairment and neuropsychiatric symptoms to be present. Almost 60% of persons with MS have neuropsychiatric symptoms, some may be present at the onset of the disease and can remain throughout its course [3]. To understand the factors that contribute to the development of these symptoms in persons with MS, this systematic review attempts to highlight and explain four main factors (morphological changes of the brain, immunological mechanism, socioeconomic aspects, and individual factors) that have recurred throughout the research process.

Morphological Changes of the Brain

Research has shown that persons with MS who experience neuropsychiatric symptoms often exhibit alterations in both grey and white matter of the brain [9]. The alterations pertain to cognitive function and the presence of mood disorders such as anxiety and depression. Cognitive function, which encompasses receiving and processing information and expressing thoughts and ideas [10] is influenced by the grey matter volumes of the brain, particularly in the frontotemporal lobes and thalamus. The degree of neurodegeneration, or atrophy, is directly related to the volume, with less volume leading to diminished cognitive function and vice versa. Interestingly, Ahmed et al. [11] found that cognitive impairment in pediatric-onset MS is also related to thalamic volume and cortical thickness of the temporal lobe. This early-onset cognitive impairment can affect later life opportunities including employment opportunities [11].

Additionally, grey matter volume tends to decrease as the disease progresses, making it a potential marker for disease progression [18]. The areas with the fastest rate of atrophy include subcortical grey matter regions such as the corpus callosum, hippocampus, amygdala, thalamus, and putamen [12]. Persons with MS who experience progressive atrophy in these regions often manifest major cognitive dysfunction in knowledge, quantitative reasoning, memory, and visual-spatial processing [13]. Moreover, Oset et al. [12] noted that cortical domains such as praxis (process) and gnosis (knowledge) are usually preserved even in the later stages of the disease, while subcortical domains are more affected. The study also indicated that cognitive decline is more prominent in progressive forms of the disease.

Another study by Adamaszek et al. [13] utilized neurophysiological tools such as event-related potentials (ERPs) and late positive potentials (LPPs) to assess neural processing. Their findings indicated that persons with MS exhibit impaired ability to discriminate various emotional characteristics, such as facial expressions, due to lesions in the prefrontal cortex; meanwhile, affective processing, like motivation, was predominantly impacted by neural damage in the anterior cingulate and amygdala-hippocampus complex. The study also highlighted that conventional MRI may not detect these irregularities in functioning and may require more advanced MRI imaging to examine the microstructural components of the white matter responsible for transmitting signals.

As for the white matter tracts, Margoni et al. [5] highlighted that lesional microstructural abnormalities in white matter tracts that are critical for cognitive function, such as the cingulum, are relevant predictors of global cognitive impairment. The study also confirmed that focal and diffuse grey matter damage, especially in the hippocampus, was significantly associated with severe cognitive dysfunction.

Looking at mood irregularities and morphological changes in MS, anxiety was initially thought to be more of a psychological reaction to the disease diagnosis [1] and not associated with any structural brain changes. However, recent research has indicated that persons with MS who experience anxiety have ventrolateral prefrontal cortex atrophy, which is dependent upon the level of severity of anxiety, and manifests as difficulty with processing threats and emotions [5].

**Figure 2:**
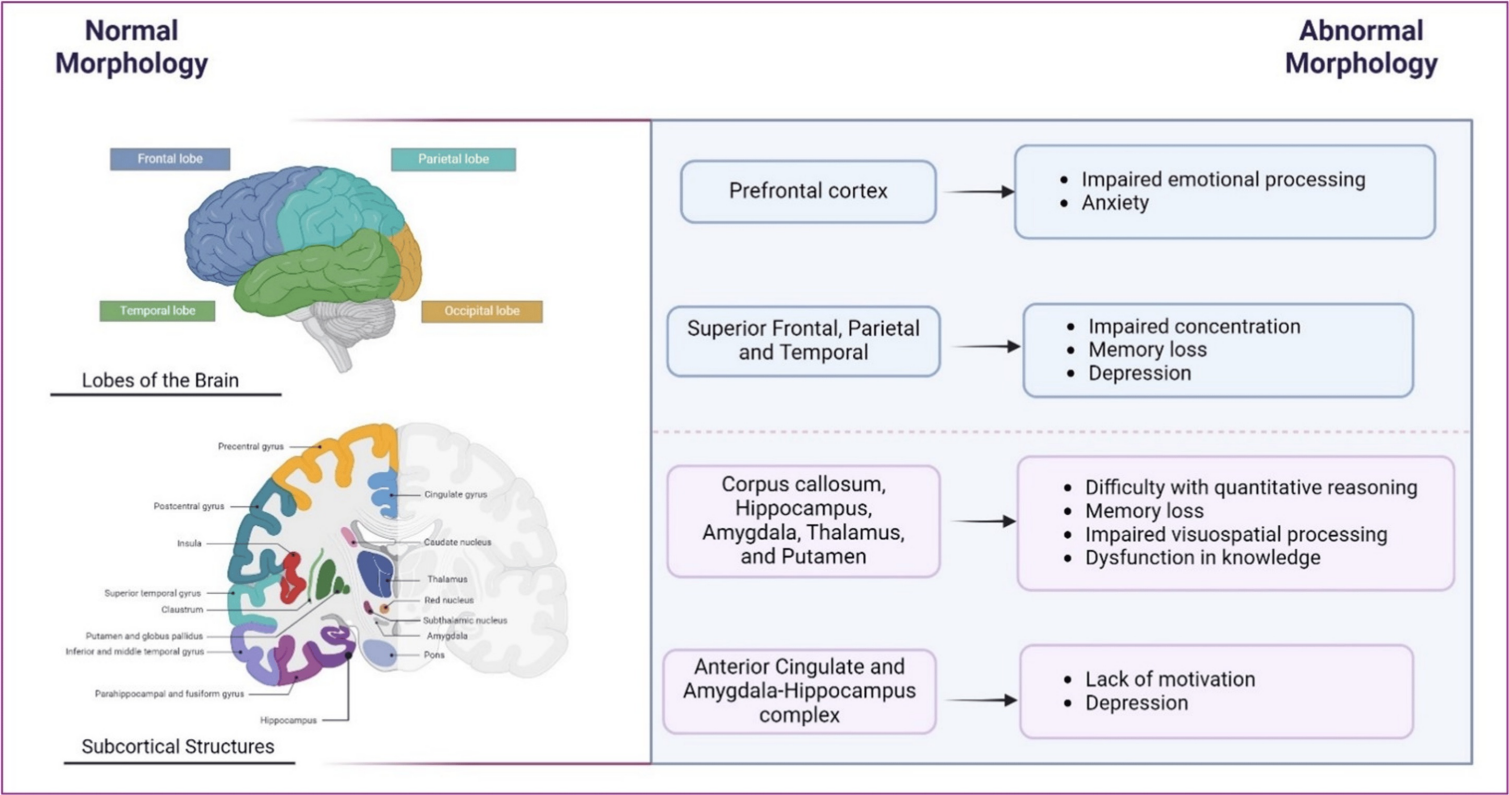
The main morphological changes and manifestations of neuropsychiatric symptoms in persons with multiple sclerosis (MS). Created in Biorender.com

While the structural changes linked to anxiety require more research, depression is directly linked to the presence of hypointense T1 lesions in the superior frontal, superior parietal, and temporal regions, as well as lateral and third ventricular dilatation and frontal atrophy, leading to impairments in concentration and memory [1]. This also increases the lifetime risk of developing major depressive disorder in MS patients by >50% [1]. Figure 2 summarizes the morphological changes and neuropsychiatric manifestations in persons with MS. 

Immunological Mechanisms

The manifestation of neuropsychiatric symptoms such as depression or major depressive disorder (MDD) in persons with MS is influenced by various immunological factors. According to Bruno et al. [9], the debate over whether depression in MS is "organic" or "reactive" emphasizes the complexities of its diagnosis. The study highlighted four main theories that attempt to explain the development of these neuropsychiatric symptoms: monoamine, neuroendocrine, neuroinflammatory, and glutamatergic theories.

The monoamine theory posits that low levels of serotonin, dopamine, and noradrenaline contribute to the onset of depressive episodes, similar to persons without MS. On the other hand, the neuroendocrine theory emphasizes dysfunction in the hypothalamic-pituitary-adrenal (HPA) axis [15], leading to increased release of corticotrophin-releasing hormone (CRH), adrenocorticotropic hormone (ACTH), and cortisol, ultimately resulting in damage to hippocampal cells and cortical neuronal atrophy.

The neuroinflammatory theory explains that persons with MS who suffer from depression have increased levels of proinflammatory cytokines, such as prostaglandins (PGs), complement (C3, C4), TNF-α, IL-1β, and IL-6, in both cerebrospinal fluid (CSF) and peripheral blood. Specifically, TNF-α and IFN-γ have been identified as inflammatory mediators that drive the severity of depressive symptoms during clinical relapses in MS. Notably, CD8+ T lymphocytes play a prominent role in explaining the development of depressive symptoms in MS. This theory also highlights the crucial role of activated microglia and infiltrating peripheral monocytes in the pathophysiology of MDD in persons with MS. Together, they cause axonal and myelin loss, and overall neurodegeneration. In addition, the glutamatergic theory suggests that the excessive release of glutamate, mediated by TNF-α, leads to the loss of post-synaptic excitatory terminals, resulting in impaired long-term potentiation (LTP) and behavioral symptoms such as memory loss and spatial disorientation. Lower levels of gamma-aminobutyric acid (GABA) have also been linked to motor disability and cognitive dysfunction in persons with MS, attributed to the release of IL-1β by autoreactive lymphocytes.

Similarly, Almulla et al. [14] highlighted the role of cytokines, primarily IL-1β, IL-6, IL-8, IL-17, IFN-γ, and TNF-α, in the development of depressive symptoms and affective dysfunction while also noting the contribution of erythron dysfunction secondary to oxidative stress triggered by the inflammatory nature of the illness.

**Figure 3:**
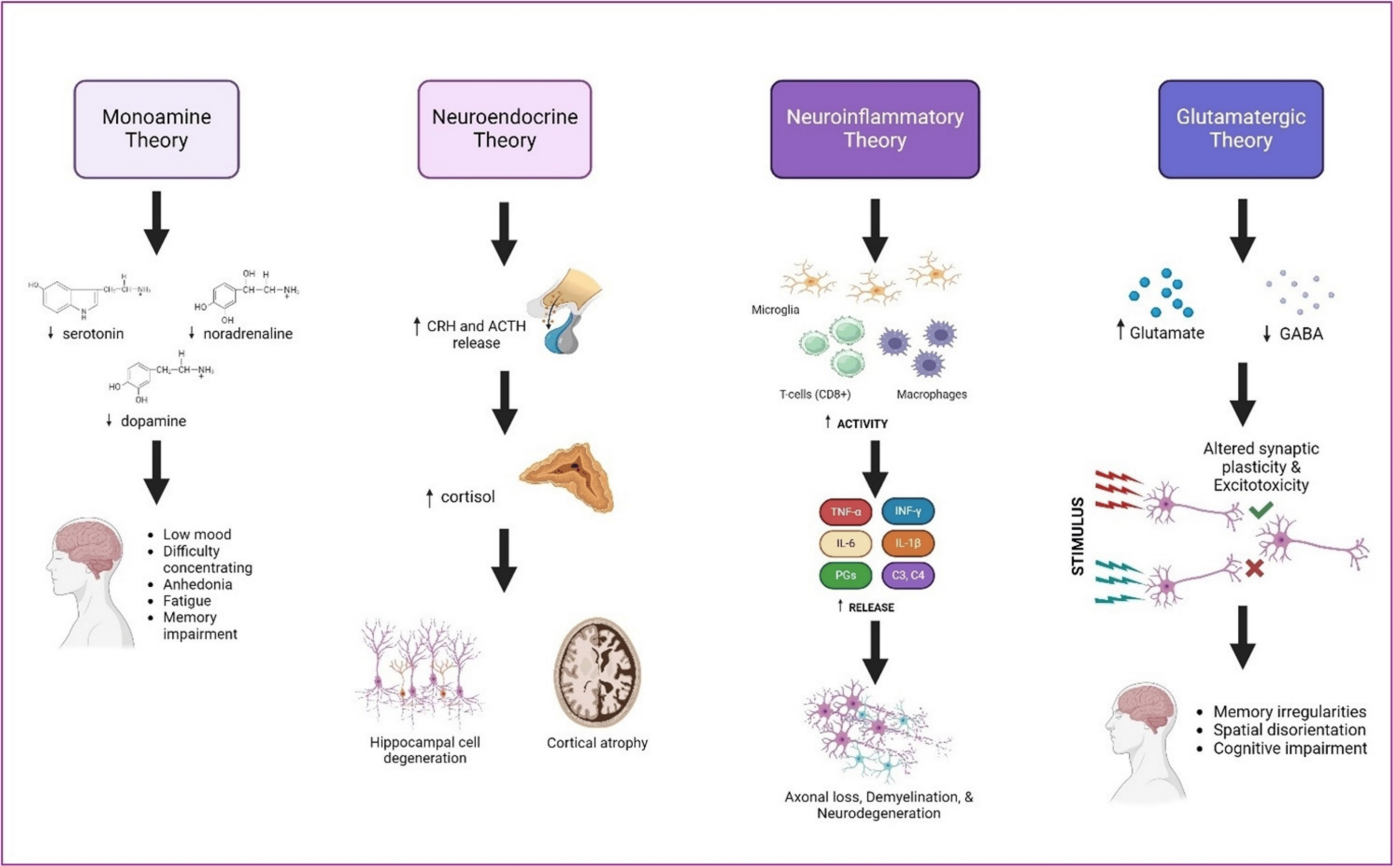
The four main immunologic mechanisms contributing to neuropsychiatric symptoms in persons with MS. CRH: corticotrophin-releasing hormone; ACTH: adrenocorticotropic hormone; GABA: gamma-aminobutyric acid; PGs: prostaglandins; C3: complement 3; C4: complement 4, IL-6: interleukin-4, IL-1β: interleukin-1 beta (); INF-ɣ: interferon-gamma Created in Biorender.com

Research in this area has shown that the immunological pathogenesis of neuropsychiatric manifestations is not limited to depression and MDD, but also extends to cases of schizophrenia and bipolar disorder [2,5]. Figure 3 highlights the four main immunological factors that contribute to the development of neuropsychiatric symptoms in persons with MS.

*Socioeconomic Factors* 

Socioeconomic factors are associated with the manifestations of neuropsychiatric symptoms. Several studies have highlighted that employment status/income, comorbidities, and access to healthcare play major roles. One study indicated that hospital admission is associated with an increased risk of developing schizophrenia and bipolar disorder in persons with MS, particularly within one year of admission [2]. Additionally, Alswat et al. [4] found that low income and high unemployment are interlinked and could potentially contribute to the prevalence of major depression and generalized anxiety disorder in persons with MS and/or are a sequela of the complications of MS. The study also had lower numbers of anxiety/depression cases reported as compared to other studies and the authors stated that the population examined may have grossly underreported their symptoms due to stigma about mental illnesses and/or assuming that their symptoms were not related to their MS diagnosis.

Additionally, socioeconomic factors can affect cognitive function in the early stages of MS, particularly concerning regular exercise, a healthy diet, managing comorbidities, and using appropriate disease-modifying therapy to stabilize or improve cognition in persons with MS [12]. Therefore, cognitive performance can be a potential indicator of disease progression and can impact the future employment status and quality of life of persons with MS [12].

Individual Factors

Multiple factors contribute to the development of neuropsychiatric symptoms in persons with MS. Oset et al. [12] highlighted that mental disorders, particularly depression and anxiety, can significantly influence cognitive abilities in persons with MS. Depression, affecting approximately 30% of persons with MS, has been associated with negative impacts on various cognitive functions, such as information processing speed, executive function, attention, and memory [12]. Similarly, anxiety disorder, which affects about 22% of persons with MS, may influence episodic memory and executive function [12]. Moreover, significant depressive symptoms were associated with worse neuroperformance, resulting in poor cognitive processing speed, lower employment rates, higher levels of fatigue, and greater physical disability, emphasizing the problematic nature of depression in persons with MS [15-17]. Vitamin D levels were also correlated with greater physical disability and higher overall relapse rates in patients with MS; however, no direct relationship was established between Vitamin D levels and neuropsychiatric manifestations in persons with MS [10].

Marrie et al. [18] found that higher Framingham risk scores (FRS), indicative of vascular risk factors such as hypertension, diabetes, and hyperlipidemia, are associated with significant loss of brain volume over time in persons with MS. Specifically, diabetes mellitus (DM) has been linked to cognitive impairment and disability progression [18]. Additionally, cognitive impairment is more prevalent in older persons with MS compared to the younger, and there is evidence suggesting that male persons with MS may have greater vulnerability to cognitive deficits compared to female persons with MS [5]. It's also important to consider lifestyle factors, as highlighted by two studies. They found that lifetime stressors, mental health issues, level of education, sleep disturbance, and smoking were all significantly associated with MS severity [19]. Resilience (in terms of coping skills) to these stressors was negatively correlated with MS severity, indicating that higher resilience scores were linked to decreased severity of MS symptoms [20,21].

Limitations

This systematic review encountered some limitations which must be considered. The search strategy included only five databases for gathering articles, and only those published in English were included. Only studies with a five-year time span (January 2020 to May 2024) that were free full texts were included, and other studies that may have had relevant information were excluded.

Additionally, the study designs utilized in this research predominantly included cross-sectional studies with small study populations (there were limited randomized-controlled trials and cohort studies available for use based on the research aim), which introduced an imbalance with the study type and, ultimately, selection bias. Furthermore, some articles did not directly address the development of neuropsychiatric symptoms in persons with MS but instead focused on the prevalence of the symptoms and their impacts on quality of life. Therefore, this systematic review recommends more comprehensive studies to be conducted with larger sample populations that focus on the factors contributing to neuropsychiatric manifestations in persons with MS.

## Conclusions

The factors described in this study, including morphological changes of the brain, immunological mechanisms, socioeconomic factors, and individual factors, collectively contribute to the development of neuropsychiatric symptoms in persons with MS. Each factor shared common overlapping features, highlighting the complexity of the pathogenesis of neuropsychiatric manifestations and the need for further research into each factor. Persons with MS experiencing neuropsychiatric symptoms exhibited alterations in grey and white matter, affecting cognitive function and mood. Cognitive impairment is related to thalamic volume and cortical thickness, while anxiety and depression are associated with specific structural brain changes. Various theories explained the development of these symptoms, including monoamine, neuroendocrine, neuroinflammatory, and glutamatergic theories. Additionally, socioeconomic and individual factors played a significant role in the manifestation of neuropsychiatric symptoms in persons with MS, including their impact on the risk of developing more serious psychiatric conditions like schizophrenia, bipolar disorder, MDD, and generalized anxiety disorder. These factors may also influence disease progression, employment status, and quality of life. Therefore, understanding and addressing these factors is essential for developing targeted interventions, improving disease management, and enhancing the overall well-being and mental health of persons living with MS.
